# To Strike a Pose: No Stereotype Backlash for Power Posing Women

**DOI:** 10.3389/fpsyg.2016.01463

**Published:** 2016-09-27

**Authors:** Miriam Rennung, Johannes Blum, Anja S. Göritz

**Affiliations:** Occupational and Consumer Psychology, Department of Psychology, Albert Ludwig University of FreiburgFreiburg, Germany

**Keywords:** power, power posing, perception, gender, warmth, competence, emotions, body language

## Abstract

Power posing, the adoption of open and powerful postures, has effects that parallel those of actual social power. This study explored the social evaluation of adopting powerful vs. powerless body postures in men and women regarding perceived warmth, competence, and the likelihood of eliciting admiration, envy, pity, and contempt. Previous findings suggest that the display of power by women may have side effects due to gender stereotyping, namely reduced warmth ratings and negative emotional reactions. An experiment (*N* = 2,473) asked participants to rate pictures of men and women who adopted high-power or low-power body postures. High-power posers were rated higher on competence, admiration, envy, and contempt compared to low-power posers, whereas the opposite was true for pity. There was no impact of power posing on perceived warmth. Contrary to expectations, the poser’s gender did not moderate any of the effects. These findings suggest that non-verbal displays of power do influence fundamental dimensions of social perception and their accompanying emotional reactions but result in comparably positive and negative evaluations for both genders.

## Introduction

Social power, defined as asymmetric control over valued resources in social relationships (Keltner et al., 2003; Magee and Galinsky, 2008), is accompanied by many positive cognitive, emotional, behavioral, and physiological consequences for the power holder (for a review, see Galinsky et al., 2015). An abundant body of research has focused on the relationship between social power and its non-verbal display. Openness and expansiveness of body posture were repeatedly identified as proximal non-verbal correlates of possessing power in both humans and animals, whereas, the lack of power is non-verbally reflected in constricted body postures (de Waal, 1998; Carney et al., 2005; Hall et al., 2005; Huang et al., 2011). This association seems to be hard-wired because the same phenomenon occurs in higher animals as well as in humans, and even congenitally blind athletes adopt power poses after a successful competition (Tracy and Matsumoto, 2008).

Recently, research has suggested that this mind-body link is bi-directional (for a review on the *embodiment*-hypothesis, see Barsalou, 2008). Not only does the experience of power influence our posture, but our posture can influence our experience of power. Specifically, *power posing*, that is, deliberately adopting an open and expansive high-power posture, induces effects in the poser that mirror the abovementioned consequences of actual social power (for a recent review, see Carney et al., 2015). For example, expansive postures were shown to parallel desirable effects of actual power in terms of enhanced abstract thinking (Huang et al., 2011), increased thought confidence (Briñol et al., 2009), better mood and self-esteem (Nair et al., 2015), more risk taking (Carney et al., 2010; Cesario and McDonald, 2013), greater action orientation (Huang et al., 2011; Park et al., 2013), improved performance in subsequent social evaluation situations (Cuddy et al., 2012, 2015), increased pain tolerance (Bohns and Wiltermuth, 2012), and more functionally adequate hormonal reactions (Carney et al., 2010; but see Ranehill et al., 2015), while reliably increasing the subjective sense of power (Carney et al., 2010; Huang et al., 2011; Park et al., 2013; Cuddy et al., 2015).

Taken together, these findings indicate power posing to be an effective tool to elicit the positive consequences that otherwise arise from actual power. Power posing used as such a tool is also quite efficient because adopting powerful postures for only a few minutes produces these effects (Carney et al., 2015). Because power poses can be adopted independent of the poser’s social role or hierarchical position, power posing seems especially suitable for persons who chronically lack power. The fact that TED talk Cuddy’s (2012, June 28) in which she highlighted power posing’s potential of improving life and career was viewed more than 25 million times suggests that a large audience deems power enhancement through body posture relevant.

### Power Posing in the Eyes of the Observer

In spite of this evidence, an unconditional recommendation of power posing may be premature. Although, much research praises the *intrapersonal* effects of power posing, we caution that there might be detrimental *interpersonal* side-effects. If power posing is displayed in a social context, it elicits social evaluations in the observer. In several real life situations, such as job interviews, dates or oral exams, these evaluations are important to the poser. However, empirical evidence about the outcomes of adopting powerful body postures in terms of interpersonal evaluations toward the power poser is scarce. In particular, little is known about the role of gender in the interpersonal perception of power posers. As evidenced by many empirical findings (for a review, see Hogue and Lord, 2007) and highlighted by current public debate (e.g., ban bossy campaign^1^), males and females in powerful positions face different expectations and evaluations. Therefore, the question arises whether these differences lead to different consequences for power-posing men and women with regard to how they are perceived by others. Answering this question would allow for tailored recommendations of how to best employ power posing as a tool. This might help level the playing field for women as well as other groups with, on average, less social power (Carli, 1999).

In the present study, we investigate both the overall impact and the gender-specific impact of adopting power-related body postures on two basic dimensions of interpersonal perception and on the emotional reactions that go along with them. In doing so, we aim to clarify whether gender-specific side effects of power posing should be taken into account when evaluating its usefulness in a real-world context.

To approach our research questions, we draw on the *stereotype content model* (SCM; Fiske et al., 2002; Cuddy et al., 2008, 2011; Caprariello et al., 2009), an established conceptual framework for the investigation of interpersonal perceptions and stereotypes, which has been validated in more than 20 cultures. The SCM extends Allport’s (1954) conceptualization of a stereotype as representing unidimensional and uniformly negative attitudes by introducing two independent dimensions: *warmth* (e.g., friendliness, warm-heartedness, kindness, empathy, benevolence) and *competence* (e.g., ability, skill, efficacy, intelligence, power). Both dimensions have been cross-culturally identified as ubiquitous and basic dimensions in the social perception of both individuals and groups (Fiske et al., 2002, 2007; Cuddy et al., 2008, 2011; Wojciszke et al., 2009). Together, these two dimensions explain up to 97% of the variance in global evaluations of individuals (Wojciszke et al., 1998). It has been theorized that evaluations on these two dimensions are fundamental for social interaction: warmth indicates whether the intentions of a social entity are positive or negative and predicts its tendency to help and support, thus facilitating a basic friend-foe distinction (Fiske et al., 2007; Cuddy et al., 2011). Competence conveys information regarding the person’s or group’s ability to successfully carry out motives and intentions (Fiske et al., 2007), thus serving as an indicator of whether fight or flight might be the better choice in case of a conflict.

Research on the antecedents of warmth and competence perceptions indicates that the structural social variables *interdependency* and *status* play a key role (Fiske et al., 2002; Cuddy et al., 2007). Whereas, interdependency (competition vs. cooperation) with the assessed social entity predicts warmth, competence is derived from information about the target’s status relative to its ingroup (Fiske et al., 2002, 2007), with average correlations between status and competence of *r* = 0.77 for individuals and *r* = 0.94 for groups (Cuddy et al., 2009). In line with the *just world belief* (Lerner and Miller, 1978), high social status (demographic variable) is attributed to high competence (personality variable). At the same time, high status signals the ability to control resources, to realize intentions, and to achieve goals (Fiske, 1993), which is consistent with our initial definition of power. Combined with the well-established connection between expansive body postures and power (Hall et al., 2005), this suggests a link between power, status, and competence at a basic level. Therefore, we expect that the display of high-power poses leads to the attribution of higher competence. In line with prior studies that show that power posing has similar effects on posing men and women when the dependent variable pertains to competence (e.g., abstraction, Huang et al., 2011; interview performance, Cuddy et al., 2012, 2015), this should hold true regardless of the poser’s gender.

*H*_1_: High-power posers are judged to be more competent than low-power posers.

Perceptions of individuals and groups in terms of their warmth and competence may also result in a variety of emotional responses in the observer. The SCM describes a systematic pattern of emotional reactions arising from the four combinations of high and low warmth and competence perceptions (e.g., Fiske et al., 2002; Cuddy et al., 2007). Accordingly, uniformly positive or negative assessments tend to elicit admiration (high warmth, high competence) or contempt (low warmth, low competence), whereas ambivalent perceptions are likely to induce envy (low warmth, high competence) or pity (high warmth, low competence). As shown above, power posing is expected to influence competence ratings. Therefore – regardless of ascribed warmth – we expect that high-power posers elicit emotions associated with high competence to a greater extent than low-power posers, whereas the opposite should be true for emotions associated with low competence.

*H*_2a_: High-power posers elicit more admiration than low-power posers.
*H*_2b_: High-power posers elicit more envy than low-power posers.
*H*_2c_: High-power posers elicit less pity than low-power posers.
*H*_2d_: High-power posers elicit less contempt than low-power posers.

### Gender-Specific Considerations

The present study aims to clarify whether there are gender-specific side effects of power posing in the interpersonal domain. Expectations concerning the nature and direction of such gender-specific backlashes can be derived from SCM-related research on the phenomenon of *ambivalent stereotyping*, which denotes holding both negative and positive attitudes toward a social entity.

In spite of their conceptual independence, warmth and competence judgments were often found to influence each other in two opposite ways (Cuddy et al., 2011). First, a *halo-effect* (Thorndike, 1920) may occur, resulting in positive correlations between both dimensions. Second, a *contrast effect* may occur, leading to negative correlations. The latter happens when surpluses in one dimension are interpreted as deficits in the other; for example, high warmth is interpreted as an indicator of low competence and vice-versa (Judd et al., 2005; Kervyn et al., 2009).

In regard to predicting gender-related side effects, it is important to note that especially women are confronted with ambivalent stereotyping. In validations of the SCM in US-American samples (Fiske et al., 1999), the majority of women was stereotyped as warm but incompetent (if they met the traditional role expectations of being housewives and mothers) or as competent but cold (if they did not meet those requirements, e.g., female leaders). Consistent with that, Eckes (2002) found that 11 out of 17 female gender-subtypes were assigned to one of the ambivalent SCM clusters, whereas this was less the case for male gender-subtypes. Furthermore, the subtype “typical woman” received high warmth and low competence ratings, whereas the “typical man”-subtype was ascribed high competence and average warmth (Eckes, 2002). In general, women are not only perceived as more warm compared to men (descriptive stereotype; Cuddy et al., 2011) but are also expected to display more warmth (prescriptive stereotype; Rudman and Glick, 1999; Abele, 2003) than men. In line with the discussed contrast effect, these elevated impressions of warmth are often accompanied – particularly in work-related contexts – by perceptions of lower competence compared to men (Cejka and Eagly, 1999; Eagly and Karau, 2002). Cuddy et al. (2004) demonstrated that working mothers elicited higher warmth than competence ratings whereas the opposite was true for working women without children. In contrast, working fathers were judged as both warm and competent, hence only women were affected by ambivalent stereotyping. Moreover, competent women in contrast to men of comparable competence suffered from decreased warmth ratings in a hiring scenario (Rudman and Glick, 1999). Furthermore, competent female leaders were more often characterized as cold compared to male leaders (Rudman and Phelan, 2008). Both men and women were found to show gender-specific ambivalent stereotyping (Heilman et al., 2004).

Because the non-verbal cues indicating warmth (such as smiling, nodding, leaning forward, or orienting the body toward others; see Cuddy et al., 2011) differ from the non-verbal cues of power, no main effect of power posing on warmth perceptions is expected. Based on the findings on gender-specific ambivalent stereotyping, however, female high-power posers might be perceived to be not only more competent but also colder than female low-power posers. Because women are more at risk than men of being subject to ambivalent stereotyping, we expect a contrast effect with female power posers in that the elevated competence ratings when adopting high-power postures come at the cost of reduced warmth ratings.

*H*_3_: Poser’s gender moderates the effect of power posing on warmth ratings, such that high-power posing women compared to men are perceived as less warm compared to low-power posers.

If women are indeed subject to stronger ambivalent stereotyping than men, this should be reflected in the pattern of emotional reactions toward female posers. The hypothesized drop in perceived warmth when adopting high-power postures should result in a relative shift of women from the admiration quadrant (high competence, high warmth) to the envy quadrant (high competence, low warmth), which should happen less with male posers.

*H*_4_: Poser’s gender moderates the effect of power posing on admiration relative to envy, such that high-power posing women compared to men elicit more envy relative to admiration compared to low-power posers.

## Materials and Methods

### Design

The study was realized as a web-based experiment using the *EFS Survey* software by QuestBack AG^2^ In a 2 (power of posture: high vs. low) × 2 (gender of poser: female vs. male) between-subjects design, participants rated pictures of either female or male persons who adopted either high-power or low-power postures. The dependent variables were the depicted person’s warmth and competence as judged by the observers, as well as the observers’ emotional reactions of admiration, envy, pity, and contempt toward the posers. We examined whether the covariates mood, arousal, and age influenced the dependent variables. Additionally, we explored if poser and participant gender interacted with regard to any of the dependent variables.

### Materials

The independent variables were manipulated via photographs that showed male and female persons in a range of high-power and low-power postures. In total, three male and three female models were photographed beforehand, each model posing in seven postures. Three postures were open and expansive high-power postures (two seated, one standing), three were closed and constricted low-power postures (two seated, one standing), and one posture was neutral. Postures were chosen based on Tiedens and Fragale (2003), Carney et al. (2010), and Cuddy et al. (2012). Each model was dressed in unostentatious clothes, that is, black shoes, black trousers, and a gray shirt. All faces were blurred to avoid confounding effects (e.g., inference of leadership quality based on facial features; Fruhen et al., 2015; van Vugt and Grabo, 2015). The photographs were shot against a green-screen that was later replaced by a white blank background. The picture size was reduced to a height of 500 pixels each with identical spacing between the edge and the model to control for differences in body size.

**FIGURE 1:**
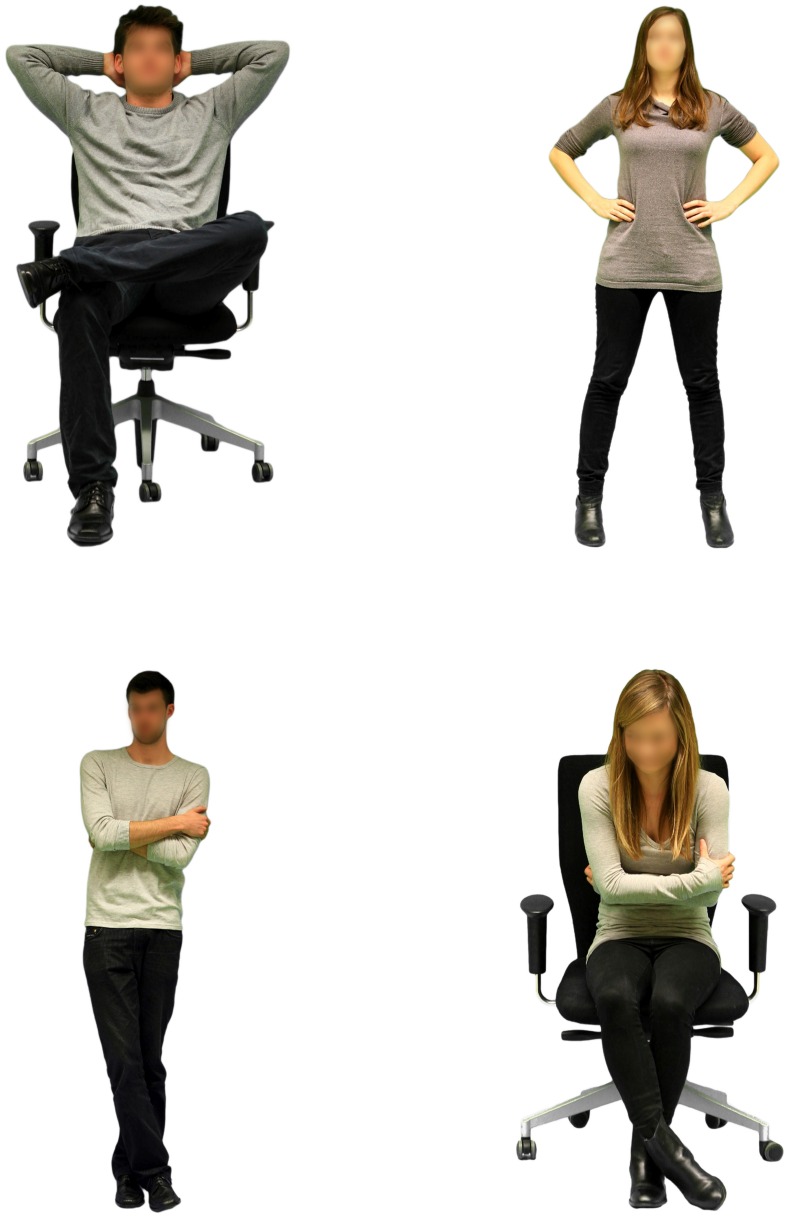
**Examples of the stimulus photographs in the four experimental conditions (**top**: high-power postures; **bottom**: low-power postures)**.

To rule out that inherent differences in apparent dominance and power among the posers affect results, we conducted a pilot study (*N* = 36). The analyses revealed that the selected models did not differ with regard to their level of perceived power when adopting a neutral posture, *F*(5,175) = 1.24, *p* = 0.29. To validate the experimental manipulation of perceived power through body posture, we piloted a second study with an independent sample (*N* = 30). The manipulation was successful: high-power postures were perceived as more powerful than low-power postures, *t*(29) = 12.46, *p* < 0.001, *d* = 2.29. Samples of high- and low-power photographs appear in **Figure 1**; for the complete stimuli, see the Supplementary Material.

### Instruments

The operationalization of the dependent variables was based on prior studies (i.e., Fiske et al., 1999, 2002; Cuddy et al., 2007; Caprariello et al., 2009; Richetin et al., 2012). Warmth was measured with the trait adjectives “warm,” “good-natured,” and “likeable”; competence was operationalized using the trait adjectives “competent,” “capable,” and “confident.” These adjectives were translated into German and validated via re-translation into English by a native speaker. The instruction for all items was to rate the extent to which the adjective described the depicted person on a six-point Likert scale (1 = *not at all*, 6 = *a lot*).

Emotional reactions were assessed by asking participants to rate the likelihood that the depicted person would elicit feelings of admiration, envy, pity, and contempt in most people, using a seven-point Likert scale (1 = *extremely unlikely*, 7 = *extremely likely*). By asking participants to take the perspective of others, we applied an indirect questioning approach. This technique increases validity for variables that are subject to social desirability (Fisher, 1993).

We used the *Self-Assessment Manikin* (SAM; Bradley and Lang, 1994) to capture participants’ current mood and arousal. The SAM uses two five-point bipolar scales to measure mood on a good–bad dimension and arousal on a calm–agitated dimension with each scale point consisting of an illustrative graphical representation of its value. The SAM is an efficient, reliable, and sufficiently valid instrument for the assessment of general affect.

### Procedure

The whole experiment followed the rules set by the ethical guidelines of the German Psychological Society (DGPs; 2004, CIII). According to the German Psychological Society’s ethical commission, approval from an institutional research board is not mandatory. Furthermore, the German Psychological Society states that research using anonymous questionnaires and causing no harm or inconvenience beyond that of day-to-day experiences is exempt from obtaining informed consent of participants. In the current online-experiment all subjects were aware of taking part in research. When signing up for the online panel, each subject was informed about the possibility of quitting online studies with no repercussions or disadvantage at any time. All participants provided informed consent and allowed us to use their collected data anonymously for publications. All data was anonymously collected and analyzed. When starting the online-experiment, participants were informed about the true aim of the study (impression formation). The online-experiment asked participants to answer questions regarding their first impression of the depicted target persons, which does not cause any harm or inconvenience beyond that of day-to-day experiences.

**FIGURE 2:**
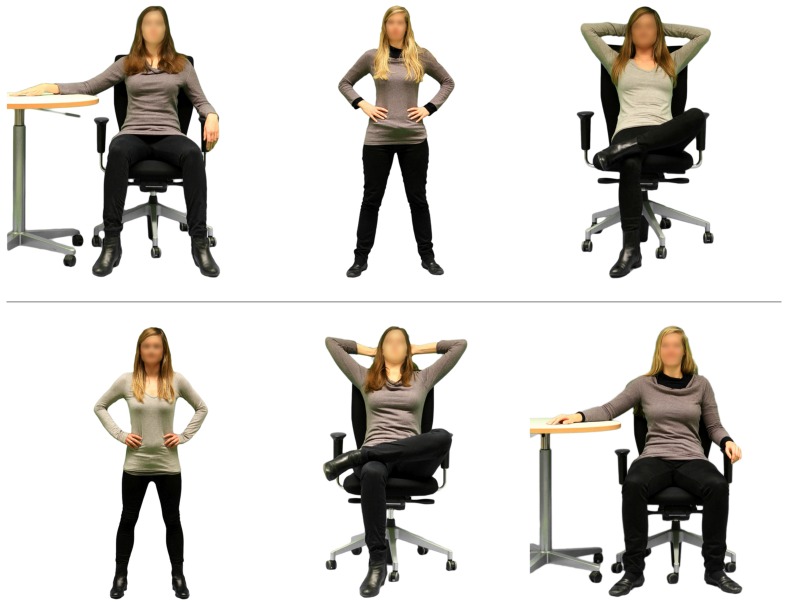
**Two sample sequences of stimulus photographs in the female high-power postures condition.** The sequence of depicted models and adopted postures (and hence the combinations of model and posture) was random.

The participants were welcomed to the study, which was announced as a study on impression formation. First, we assessed mood and arousal via the SAM. The participants were then randomly assigned to one out of four conditions. In each condition, participants observed and rated three photographs, which consisted of three different models of the same gender who adopted three different postures of the same power condition (either low-power or high-power, see **Figure 2** for an example). The sequences of models and postures were completely randomized, that is, each model of either gender condition could be displayed in each posture at each position. To ensure that participants based their ratings on their first impression of the depicted models, we displayed each photograph for 15 s. After each photograph, we asked participants to evaluate the person they had just observed with regard to the dependent variables. In the end, we asked participants to indicate what they thought the study was about (i.e., suspicion check).

### Participants

We recruited participants via WiSoPanel^3^, a non-commercial online access panel that holds people from all walks of life (Göritz, 2014). A total of 2,634 participants completed the survey in one session. We eliminated 41 participants because they had participated using a smartphone in spite of instructions not to do so or because they had reported technical difficulties. Another 120 participants were excluded whose duration for one of the three rating tasks was below the 1st percentile (Task 1: 24.73 s; Task 2: 19.97 s; Task 3: 17.81 s) or above the 99th percentile (Task 1: 349.23 s; Task 2: 386.59 s; Task 3: 386.88 s).

This procedure left 2,473 participants (57.1% women) in the analyses. The mean age was 48.6 years (*SD* = 14.5). In the sample, 34.2% had a college degree or higher, another 25.7% had a high school degree or equivalent, 27.9% had a secondary school certificate/10th-grade degree, 11.2% had a basic school qualification/ninth-grade degree, and 1.0% had not (yet) graduated from school. Most participants (59.4%) were employed when they signed up for the panel, 14.8% were in retirement, another 13.6% were studying or in vocational training, 5.9% were unemployed, 1.2% were in parental leave, and 5.1% did not categorize themselves into one of these categories.

### Analyses

Warmth and competence ratings were processed by averaging the three item means, which in turn were obtained by averaging all three rating tasks within each participant. The internal consistency of the competence ratings was α = 0.84, and the warmth ratings had an internal consistency of α = 0.92. For the purpose of testing *H*_4_, we computed relative values by subtracting the envy scores from the admiration scores.

To test the hypotheses, we conducted a set of two-factorial analyses of variance (ANOVA). The effects of power (high vs. low), posers’ gender (male vs. female) and their interaction were assessed separately for each dependent variable.

Scale means, standard deviations, and intercorrelations can be found in **Table 1.** Because the amount of shared variance between the dependent variables and the proposed covariates of mood, arousal, and age was consistently small (all *R*^2^ < 0.04) or non-significant, we decided to not include those covariates into the statistical models. Furthermore, there were no significant interactions between the posers’ gender and the participants’ gender for any of the dependent variables (all *p*s ≥ 0.22). Therefore, in favor of more robust statistical models, we did not consider the participants’ gender in the following analyses.

**Table 1:** Variable means, standard deviations, and intercorrelations.

	Variable	*M*	*SD*	1	2	3	4	5	6	7	8	9	10
1	Warmth	3.40	0.78	1									
2	Competence	3.53	0.86	0.59^∗∗^	1								
3	Admiration	3.19	1.21	0.47^∗∗^	0.69^∗∗^	1							
4	Envy	2.90	1.21	0.22^∗∗^	0.51^∗∗^	0.71^∗∗^	1						
5	Pity	3.53	1.22	0.29^∗∗^	-0.15^∗∗^	0.03	-0.02	1					
6	Contempt	2.88	1.18	-0.15^∗∗^	0.04^∗^	0.17^∗∗^	0.42^∗∗^	0.08^∗∗^	1				
7	Mood	3.52	0.85	0.07^∗∗^	0.03	0.04	0.00	0.04	-0.03	1			
8	Arousal	2.08	0.94	0.03	0.04^∗^	0.07^∗∗^	0.10^∗∗^	0.05^∗∗^	0.08^∗∗^	-0.34^∗∗^	1		
9	Age	48.61	14.45	-0.02	-0.04^∗^	-0.04	-0.10^∗∗^	0.01	-0.19^∗∗^	0.07^∗∗^	-0.17^∗∗^	1	
10	Gender	1.43	0.50	0.01	-0.01	0.04	0.03	0.02	0.02	0.08^∗∗^	-0.07^∗∗^	0.20^∗∗^	1


*N* = 2,473 for all variables. *p < 0.05; **p < 0.01.

## Results

### Competence

**FIGURE 3:**
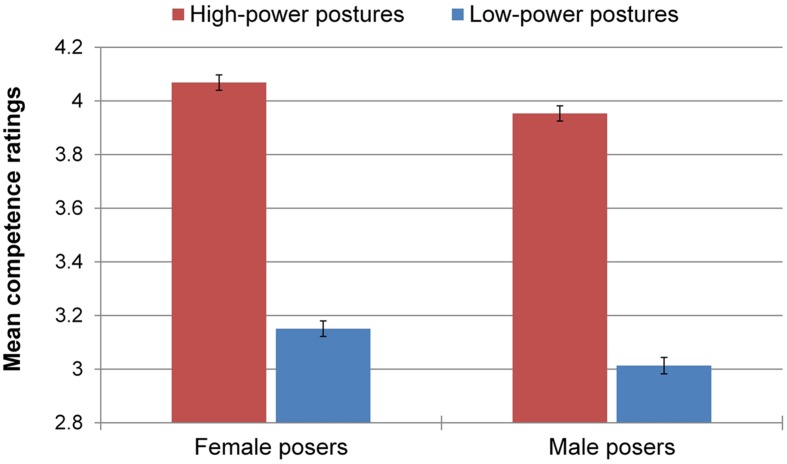
**Mean competence ratings by experimental condition.** Error bars represent ± 1 standard error of the mean (SEM).

There was a significant main effect of power, *F*(1,2469) = 1006.89, *p* < 0.001, $\eta_{p}^{2}$ = 0.290, 90% CI [0.266; 0.313]. Participants assigned higher competence ratings to high-power posers (*M* = 4.01, *SD* = 0.70) compared to low-power posers (*M* = 3.08, *SD* = 0.75). Therefore, *H*_1_ was upheld. Additionally, there was a small but significant main effect of gender, *F*(1,2469) = 18.70, *p* < 0.001, $\eta_{p}^{2}$ = 0.008, 90% CI [0.003; 0.014], with participants granting slightly higher competence ratings to female posers (*M* = 3.59, *SD* = 0.86) than to male posers (*M* = 3.48, *SD* = 0.87). As expected, the Power × Gender interaction was not significant, *F*(1,2469) = 0.15, *p* = 0.70; hence, the tendency to rate high-power posers as more competent was independent of poser’s gender (**Figure 3**).

### Warmth

**FIGURE 4:**
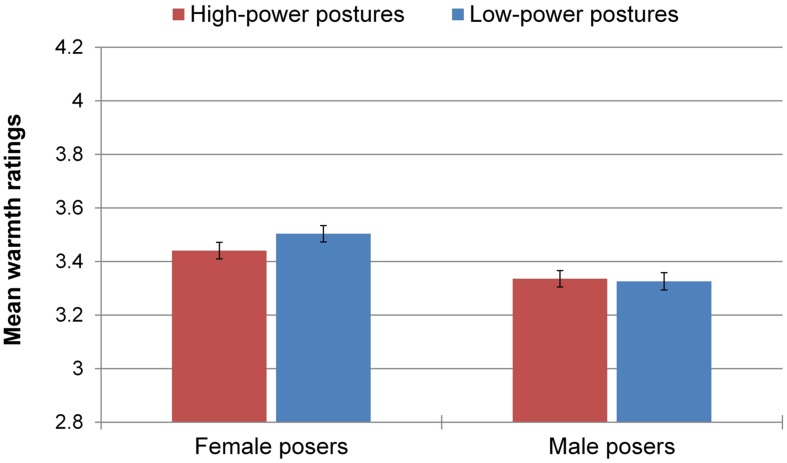
**Mean warmth ratings by experimental condition.** Error bars represent ± 1 SEM.

There was no main effect of power, *F*(1,2469) = 0.74, *p* = 0.39, but there was a small main effect of poser’s gender on perceived warmth, *F*(1,2469) = 20.59, *p* < 0.001, $\eta_{p}^{2}$ = 0.008, 90% CI [0.003; 0.015], with participants evaluating female posers as being slightly warmer (*M* = 3.47, *SD* = 0.78) than male posers (*M* = 3.33, *SD* = 0.77). Contrary to *H*_3_, the Power × Gender interaction did not reach statistical significance, *F*(1,2469) = 1.36, *p* = 0.24 (**Figure 4**).

### Emotional Reactions

**FIGURE 5:**
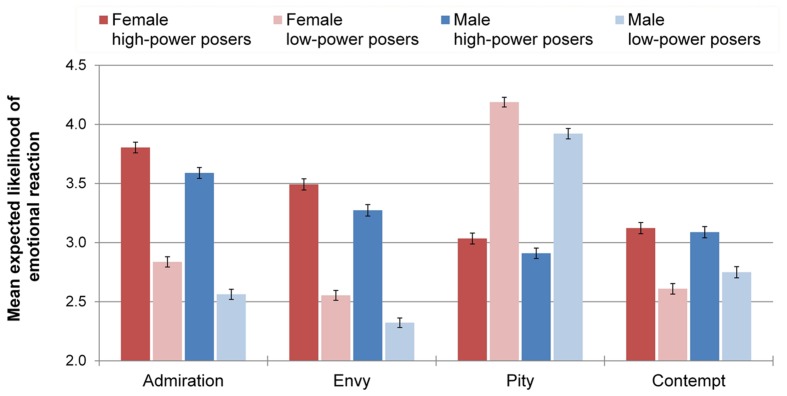
**Mean emotion ratings by experimental condition.** Error bars represent ± 1 SEM.

The analyses indicated a pattern across the four emotions of large main effects of power, small main effects of gender, and no interactions (**Figure 5**). Accordingly, a MANOVA confirmed a significant main effect of power on emotions, Pillai’s trace = 0.40, *F*(4,2466) = 410.63, *p* < 0.001, $\eta_{p}^{2}$ = 0.400, 90% CI [0.376; 0.421], a significant main effect of gender on emotions, Pillai’s trace = 0.02, *F*(4,2466) = 13.98, *p* < 0.001, $\eta_{p}^{2}$ = 0.022, 90% CI [0.013; 0.031], but no significant Power × Gender interaction, Pillai’s trace = 0.00, *F*(4,2466) = 2.02, *p* = 0.09.

#### Admiration

Confirming *H*_2a_, the ANOVA indicated a main effect of power on admiration, *F*(1,2469) = 504.74, *p* < 0.001, $\eta_{p}^{2}$ = 0.170, 90% CI [0.148; 0.192], with high-power posers (*M* = 3.70, *SD* = 1.12) receiving higher admiration ratings compared to low-power posers (*M* = 2.70, *SD* = 1.10). Furthermore, a small main effect of gender occurred, *F*(1,2469) = 30.47, *p* < 0.001, $\eta_{p}^{2}$ = 0.012, 90% CI [0.006; 0.020], with female posers (*M* = 3.30, *SD* = 1.21) being more admired than male posers (*M* = 3.07, *SD* = 1.21). There was no Power × Gender interaction, *F*(1,2469) = 0.45, *p* = 0.50.

#### Envy

In line with *H*_2b_, there was a main effect of power on envy, *F*(1,2469) = 447.96, *p* < 0.001, $\eta_{p}^{2}$ = 0.154, 90% CI [0.133; 0.175], with high-power posers (*M* = 3.38, *SD* = 1.18) receiving higher envy ratings than low-power posers (*M* = 2.44, *SD* = 1.05). In addition, gender had a small main effect, *F*(1,2469) = 25.56, *p* < 0.001, $\eta_{p}^{2}$ = 0.010, 90% CI [0.005; 0.018], in that female posers (*M* = 3.00, *SD* = 1.22) were met with more envy than male posers (*M* = 2.79, *SD* = 1.19). There was no Power × Gender interaction, *F*(1,2469) = 0.02, *p* = 0.89.

Concerning the difference scores between admiration and envy, there was no main effect of power, *F*(1,2469) = 1.99, *p* = 0.16, and no main effect of poser’s gender, *F*(1,2469) = 0.27, *p* = 0.60. In particular, there was no Power × Gender interaction, *F*(1,2469) = 0.39, *p* = 0.53, thus rejecting *H*_4_.

#### Pity

Consistent with *H*_2c_, there was a main effect of power on pity, *F*(1,2469) = 610.65, *p* < 0.001, $\eta_{p}^{2}$ = 0.198, 90% CI [0.176; 0.221], with high-power posers (*M* = 2.97, *SD* = 1.11) eliciting less pity than low-power posers (*M* = 4.06, *SD* = 1.08). Furthermore, gender exerted a small main effect on pity, *F*(1,2469) = 20.00, *p* < 0.001, $\eta_{p}^{2}$ = 0.008, 90% CI [0.003; 0.015], with female posers (*M* = 3.64, *SD* = 1.23) eliciting more pity than male posers (*M* = 3.42, *SD* = 1.20). The Power × Gender interaction was non-significant, *F*(1,2469) = 2.65, *p* = 0.10.

#### Contempt

There was a main effect of power on contempt, *F*(1,2469) = 83.64, *p* < 0.001, $\eta_{p}^{2}$ = 0.033, 90% CI [0.022; 0.045]. Contrary to predictions, the direction of the effect was such that high-power posers (*M* = 3.11, *SD* = 1.16) elicited higher contempt ratings than low-power posers (*M* = 2.68, *SD* = 1.16). Therefore, *H*_2d_ was rejected. There was no main effect of gender, *F*(1,2469) = 1.29, *p* = 0.26, and no significant Power × Gender interaction, *F*(1,2469) = 3.51, *p* = 0.06.

## Discussion

The goal of the present study was to investigate the overall impact and the gender-specific impact of adopting dominant vs. submissive body postures on perceived warmth, competence, and their accompanying emotional reactions of admiration, envy, pity, and contempt. In a large and heterogeneous sample of 2,473 participants, we found that power posing influenced assessments of the posers with regard to all dependent variables except warmth.

### Overall Impact of Power Posing

As predicted in *H*_1_, participants judged high-power posers to be substantially more competent than low-power posers. This is in line with prior studies that concluded that competence is linked to status and power (Fiske, 1993; Fiske et al., 2002, 2007), whereby status and power in turn are inherently linked to dominant body language (Hall et al., 2005). Because we examined the effect of power-related body language isolated from other cues that conveyed competence-related information (e.g., occupation, social status, role, actual behavior), our results confirm the literature by demonstrating that information derived from visible body posture alone considerably influences competence perceptions.

Furthermore, we found that adopting high-power postures as opposed to low-power postures resulted in specific emotional reactions toward the poser. As predicted, high-power posers were more likely to elicit admiration (*H*_2a_) as well as envy (*H*_2b_) and less likely to elicit pity (*H*_2c_). Together with our finding that power posing leads to higher competence ratings (*H*_1_), this supports SCM (Fiske et al., 2002; Cuddy et al., 2007). Contrary to predictions, however, high-power posers were more likely to elicit contempt than low-power posers. Interestingly this finding dovetails with Caprariello et al. (2009), who also failed to confirm SCM’s predictions only with regard to contempt, thus making this special and intensely negative emotion a fruitful candidate for future investigation.

### Gender-Specific Impact of Power Posing

Contrary to expectations as derived from gender-specific ambivalent stereotyping, no reduction of perceived warmth when women adopted high-power poses compared to low-power poses occurred, despite the substantial increase in perceived competence. Therefore, *H*_3_ was rejected. Research indicates that contrast effects such as ambivalent stereotyping of women are more likely to occur in competitive contexts, which involve comparisons between persons, than in non-competitive contexts (Judd et al., 2005). Although participants rated three different posers in our experiment, the facts that the posers were assessed one after the other and instructions did not require participants to compare the posers could have prevented a contrast effect. Warmth ratings were independent of competence ratings, which is in line with warmth and competence being two independent dimensions (Cuddy et al., 2008).

This finding fails to support the feared stereotype backlash for women that they might be punished with reduced warmth ratings when overtly displaying competence through open and expansive body postures. We consider this to be an encouraging finding – especially for women, who are supposedly more at risk to be subject to ambivalent stereotyping. This notion is further supported because the dreaded gender-specific backlash was again not found on the level of ensuing emotional reactions, as *H*_4_ was rejected as well. In fact, the pattern of how power posing influenced all four emotional reactions was the same for male and female posers. Furthermore, the poser’s gender did not moderate the positive impact of power posing on perceived competence as well. These findings dovetail with Carney et al. (2005), who found that participants attribute the same non-verbal behaviors to powerful men and women. Our study extends these findings, by showing that powerful men and women are also met with the same emotional reactions and ascriptions of warmth and competence. Statistical analyses revealed a series of main effects of poser’s gender. Female posers were perceived as both slightly more competent and slightly warmer than their male counterparts, irrespective of their posture. These differences were reflected in an SCM-consistent way on the level of emotional reactions, with female posers eliciting somewhat more admiration, envy, and pity than male posers. However, all of these gender effects were small (all $\eta_{p}^{2}$ ≤ 0.012). Perhaps, the gender difference in rated competence reflects a maturity effect (i.e., faster biological maturation of young women than men against the background that all of our posers were not older than 30) and/or a small gender-specific age difference in our stimuli (average age: 27.7 years for female posers, 25 years for male posers). The gender differences in rated warmth mirror the widespread stereotype of women being warmer than men (see Cuddy et al., 2011).

### Implications

Our findings show that power posing leads to both positive and negative consequences in the interpersonal perception of the poser. Adopting a powerful pose substantially increased the poser’s perceived competence and his or her likelihood of being admired. This indicates that power posing may benefit the poser in real-life social evaluation situations where strategic impression management is important, such as job interviews, assessment centers, negotiations, and sales pitches. Thus, together with Cuddy et al. (2012, 2015) findings that power posing may be used as a performance-enhancing preparation before a high-stakes social evaluation, the results at hand underline power posing’s usefulness because it seems to be a useful tool not only *prior* to a high-stakes social evaluation situation but also when employed *during* such a situation.

At the same time, however, overtly displaying power through non-verbal behavior elicited envy and contempt, whereas it dampened pity. It does not come as a surprise that powerful and competent people receive more envy and little pity, given their desirable position. As a high-power poser, one might not obtain one (i.e., ascribed competence) without the other (i.e., reactions of above-average envy and below-average pity). Because envy and pity predict both positive and negative behaviors toward the target (envy: passive association and active harm; pity: active helping and passive neglect; Cuddy et al., 2007; Becker and Asbrock, 2012), this is not only a downside. If high-power posers are aware of those effects, they may influence the situation to their advantage. The increased likelihood of eliciting contempt is an unexpected – and for any high-power poser – undesirable outcome, given that contempt predicts behaviors of active harm and passive neglect (Cuddy et al., 2007). Because contempt ratings were predominately located in the *unlikely* range of the scale even in the high-power conditions, elicited contempt may not be a major concern though. It is important to note that we did not find any gender-specific backlashes of power posing; thus, the outlined consequences of power posing hold equally true for men and women. Although, it is plausible that gender-related expectations still play a role when factors such as context, roles or existing hierarchies are salient, observers’ assessments at this basic perceptional level were independent of the poser’s and their own gender. We therefore conclude that women should feel encouraged by these findings to embrace the benefits of strategically demonstrating power and competence in manifold social situations.

### Limitations and Future Research

Our conclusions are based on robust effects from a large and diverse sample that took part in a true experiment. We pretested the stimulus materials, statistically controlled for potentially confounding variables and applied control techniques to exclude participants who supposedly did not answer seriously. Nevertheless, the interpretation of our data is subject to some limitations.

With regard to the manipulation of the independent variables, it should be noted that all of our posers were light-skinned persons between 23 and 30 years of age. They were evaluated by a predominantly light-skinned sample but with a wider age span. As long as we do not have data on posers as well as participants of other ethnicities and in different age spans, generalizations of our findings to other populations should be evaluated critically. In a similar vein, variation in our stimulus material was restricted to three powerful and three powerless body postures that varied in the extent of openness and self-touch. Although, openness of body posture and self-touch were repeatedly found to indicate the extent of power (for a review see Hall et al., 2005), they represent only a subset of the non-verbal behaviors associated with power. For example, interpersonal distance, other-touch or voice and speech characteristics were also associated with the extent of power in prior studies (Hall et al., 2005). Although, we consider it likely that these other non-verbal behaviors would have elicited similar reactions in participants, future research is needed to probe the generalizability of our results to other non-verbal correlates of power. Furthermore, while the depicted static photographs with blurred-faced posers, neutral clothing, and scarcity of contextual information allowed for a high level of standardization and isolation of the effects of power posing, this operationalization comes at the expense of reduced ecological validity. Consequently, generalizability to dynamic real-life situations is to be explored further. It remains unclear whether in real and dynamic interactions factors such as clothing, facial expression, behavior, roles, or setting would superimpose or interact with the effects of power posing on the observer. For example, if the poser’s outward behavior is at odds with his or her actual status, power posing might be judged as arrogant or presumptuous. Consequently, engaging in power posing in a social interaction when interaction partners consider it inappropriate may elicit the same negative reactions common to disconfirming expectations in general: resentment, stereotyping, and even behavioral discrimination (Rudman, 1998; Anderson et al., 2008; Magee and Galinsky, 2008).

Moreover, it might make a difference if the power poser is aware of the observers’ potential reactions and expectations. For example, Rucker et al. (2014) found that the effects of power on power posers’ information processing were influenced by whether posers focused on the intrapersonal experience of power or on the interpersonal expectations toward the powerful. When focused on the intrapersonal experience of power, low-power participants engaged in greater information processing than high-power participants. When focused on interpersonal expectations toward the powerful, high-power participants engaged in greater information processing compared to low-power participants, which is consistent with common expectations people have for individuals in powerful positions. Taken one step further, the awareness about other’s expectations may not only be relevant in social interactions, but may impact the power poser even devoid of any social interaction (e.g., in the case of preparatory power posing) – depending on the power poser’s focus. This highlights the importance to better understand the interpersonal dynamics associated with power posing.

Given that often times stereotyping is revealed in subtle ways, it is possible that the explicit measures employed in the current research are not sensitive to the sorts of backlash that might occur (e.g., more subtle changes in language, social behavior, etc.). Thus, future research may want to replicate our findings using a more naturalistic setting and applying implicit instead of explicit measures.

Furthermore, we measured warmth and competence with three items each, and each of the four emotional reactions with only one item. Although, this yielded good internal consistencies and was a necessary tradeoff to limit the time span for which participants needed to memorize the photograph depicted beforehand, one might argue that our approach does not capture the entirety of the complex constructs in question. Future studies could address this issue, for example, by assessing either only warmth and competence or only the emotional reactions, which in turn would allow for an increased number of items to measure these constructs.

Finally, although it is good news that this highly powered experiment did not indicate any gender-specific backlash of high-power posing, this might not universally be the case. Our sample of predominantly Germans are from a Western country that has a relatively equal status of men and women and relatively high egalitarian gender ideals (Tesch-Römer et al., 2008). Therefore, this particular result might not replicate in other countries or even cultures. It would be interesting to examine whether in cultures with lower egalitarian status of women power posing women are punished in that they are perceived comparatively colder.

## Conclusion

While an ever growing body of research has investigated the effect of power posing on the actor, this study is the first to explore how power posers are perceived. The present study complements current literature that demonstrated that power posing serves as an effective and efficient tool to induce favorable psychological states in the poser by demonstrating that power posing bears a great potential for strategic impression management. Adopting open and expansive high-power postures resulted in considerably higher perceptions of competence and a higher likelihood of being admired, and these positive outcomes did not come at the expense of reduced warmth ratings. However, power posers also faced an increased likelihood of eliciting envy and contempt and a reduced probability of being pitied. Therefore, based on situational constraints and desired outcomes, posers should carefully consider whether the advantages outweigh the downsides before engaging in power posing in the presence of observers. Because no gender-specific advantages or backlashes of power posing occurred, our results indicate that both men and women can benefit equally from non-verbal displays of power.

## Author Contributions

MR, JB, and AG developed the study concept and design. AG collected the data. MR and JB performed the data analyses. JB drafted an initial version of the manuscript. MR and AG provided revisions. All authors approved the final version of the manuscript for submission. MR and JB contributed equally to this work and share first authorship.

## Conflict of Interest Statement

The authors declare that the research was conducted in the absence of any commercial or financial relationships that could be construed as a potential conflict of interest.

## Footnotes

1 http://www.banbossy.com

2 http://www.unipark.info.

3 www.wisopanel.net
