# Supplementary material for: To Strike a Pose: No Stereotype Backlash for Power Posing Women
Source: Front Psychol. 2016 Sep 27;7:1463. doi: 10.3389/fpsyg.2016.01463 (PMC5037219; doi:10.3389/fpsyg.2016.01463)
Supplement: Supplementary file 1 [file Data_Sheet_1.DOCX]

Supplementary Material

To Strike a Pose: No Stereotype Backlash for Power Posing Women

Miriam Rennung^*^, Johannes Blum, Anja S. Göritz

*** Correspondence:** Miriam Rennung: miriam.rennung@psychologie.uni-freiburg.de

| **Model** | **Posture 1** | **Posture 2** | **Posture 3** |
| --- | --- | --- | --- |
| **w1**  26 years  171 cm | 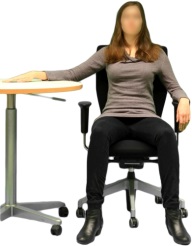 | 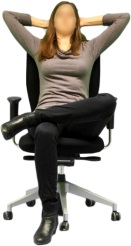 | 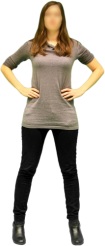 |
| **w2**  30 years  177 cm | 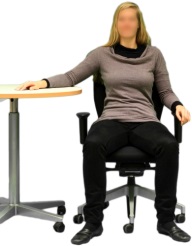 | 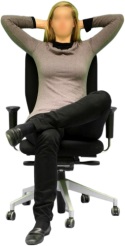 | 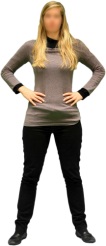 |
| **w3**  26 years  159 cm | 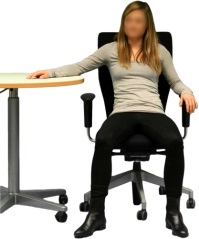 | 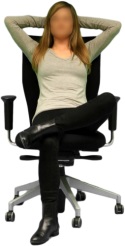 | 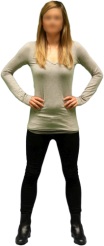 |

**Supplementary Figure 1.** Photographs of the female posers in high-power body postures.

| **Model** | **Posture 1** | **Posture 2** | **Posture 3** |
| --- | --- | --- | --- |
| **w1**  26 years  171 cm | 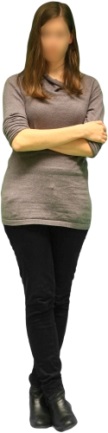 | 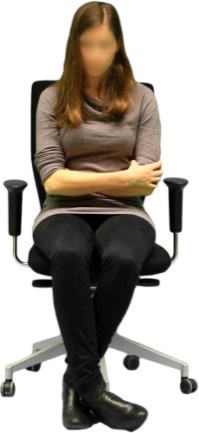 | 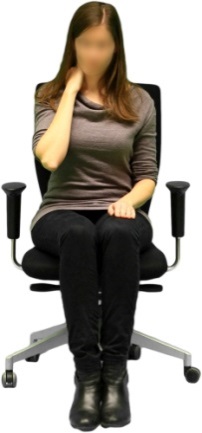 |
| **w2**  30 years  177 cm | 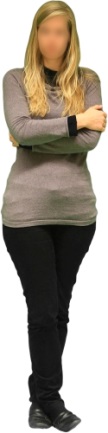 | 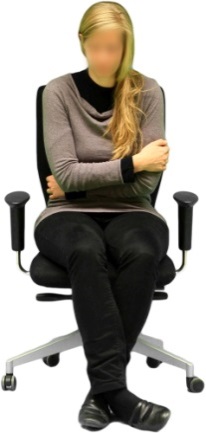 | 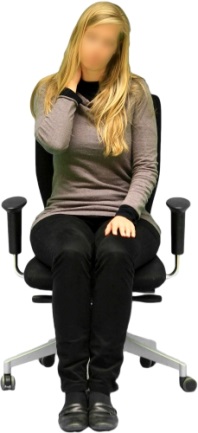 |
| **w3**  26 years  159 cm | 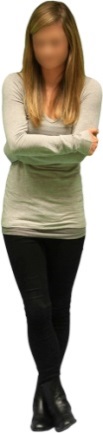 | 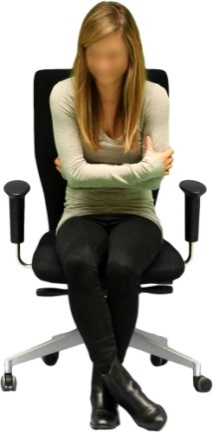 | 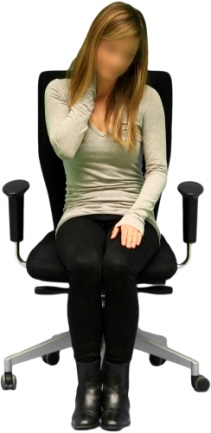 |

**Supplementary Figure 2.** Photographs of the female posers in low-power body postures.

| **Model** | **Posture 1** | **Posture 2** | **Posture 3** |
| --- | --- | --- | --- |
| **m1**  23 years  185 cm | 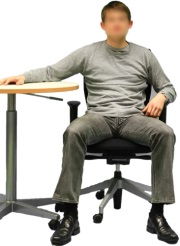 | 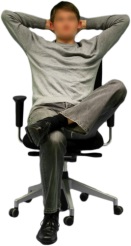 | 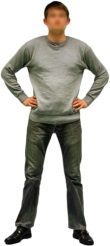 |
| **m2**  25 years  196 cm | 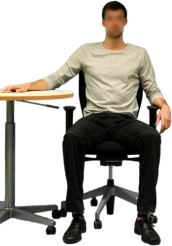 | 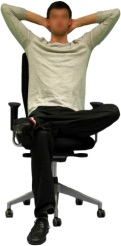 | 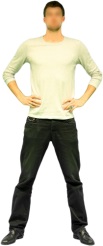 |
| **m3**  27 years  180 cm | 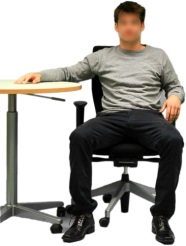 | 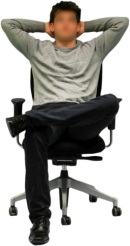 | 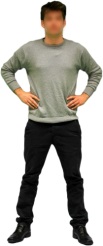 |

**Supplementary Figure 3.** Photographs of the male posers in high-power body postures.

| **Model** | **Posture 1** | **Posture 2** | **Posture 3** |
| --- | --- | --- | --- |
| **m1**  23 years  185 cm | 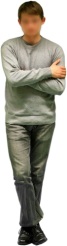 | 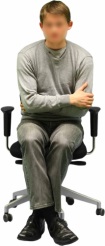 | 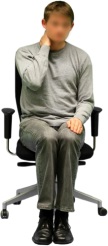 |
| **m2**  25 years  196 cm | 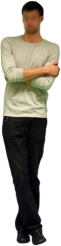 | 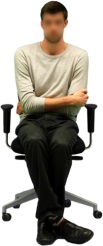 | 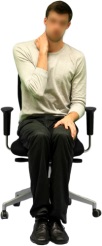 |
| **m3**  27 years  180 cm | 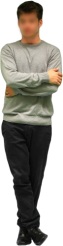 | 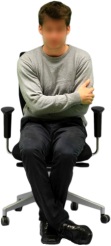 | 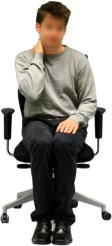 |

**Supplementary Figure 4.** Photographs of the male posers in low-power body postures.
